# York-Tank: A Degradation-Aware and Difficulty-Stratified Stereo Dataset for Underwater Visual SLAM

**DOI:** 10.3390/s26206567

**Published:** 2026-10-16

**Authors:** Chengdong Zhu, Mark Post

**Affiliations:** Department of Electronic Engineering, University of York, Heslington, York YO10 5DD, UK; chengdong.zhu@york.ac.uk

**Keywords:** underwater visual SLAM, stereo dataset, dominant degradation factor, difficulty-stratified evaluation, 6-DoF reference trajectory

## Abstract

Coupled visual degradations in underwater recordings make it difficult to relate simultaneous localization and mapping (SLAM) performance changes to specific factors and their severity. We present York-Tank, which is a controlled stereo dataset combining dominant-factor sequence design with within-factor severity stratification. Its 16 sequences contain 18,948 synchronized stereo image pairs covering low illumination, non-uniform illumination, fast motion, texture deficiency, and dynamic interference. The dataset includes baseline sequences, easy and hard conditions within each degradation category, and hybrid sequences combining multiple degradations. Preset acquisition settings and post-acquisition image and motion indicators characterize the recorded degradation severity. York-Tank-GT provides six-degree-of-freedom reference trajectories using a single overhead camera observing an above-water AprilTag array rigidly connected to the robot without directly relying on degraded underwater stereo images. Evaluations of ORB-SLAM3 and SVO Pro reveal system-dependent responses to the same degradation conditions. Controlled front-end comparisons within ORB-SLAM3 show that the effects of contrast-limited adaptive histogram equalization and dynamic-region feature rejection vary with degradation type and severity. York-Tank supports the targeted evaluation of degradation sensitivity and front-end interventions, complementing field-collected underwater datasets.

## 1. Introduction

Underwater visual simultaneous localization and mapping (SLAM) is an important enabling technology for autonomous navigation and environmental perception by underwater robots with applications in marine exploration, infrastructure inspection, and environmental monitoring [1]. Recent research on coordinated marine operations includes information-collection planning under underwater communication constraints [2] and event-driven cooperative localization and formation control for multiple unmanned surface vessels [3]. Underwater visual observations, however, are vulnerable to illumination degradation, fast motion, texture deficiency, and dynamic interference. Low illumination reduces visible detail and effective contrast, whereas non-uniform illumination produces spatially uneven brightness. Fast motion can introduce motion blur, while texture-deficient regions and dynamic objects reduce the number of stable and repeatable feature observations. These degradations affect feature detection, feature matching, and pose estimation through different mechanisms, ultimately increasing the localization error or interrupting tracking [1,4]. Existing studies have accordingly used image enhancement, robust feature extraction and matching, and dynamic-region processing to improve the robustness of underwater visual SLAM under specific degradation conditions [5,6,7]. Evaluation data should therefore do more than compare the overall performance of different methods on a given sequence. They should also reveal how performance changes relate to specific degradation factors and characterize performance decline and failure as degradation severity increases.

Existing underwater visual datasets can be broadly divided into those collected in real environments and those acquired under controlled conditions. Datasets such as AQUALOC, Caves, and Eiffel Tower cover natural marine environments, underwater caves, and deep-sea structures, providing an important basis for SLAM evaluation, three-dimensional reconstruction, and long-term observation in complex underwater settings [8,9,10]. Controlled tank datasets such as HAUD and TANK instead improve experimental repeatability and quantitative evaluation through relatively stable acquisition environments and reference-trajectory systems [11,12]. TANK further defines easy, medium, and hard difficulty levels, but each level jointly changes several conditions, including motion speed, illumination, operating range, and visible scene structure. Existing datasets therefore offer complementary strengths in environmental realism, acquisition repeatability, and reference-trajectory provision, and they support overall comparisons among SLAM methods.

When a difficulty level is defined by simultaneous changes in several acquisition conditions, however, cross-level comparisons mainly reflect the aggregate effect of coupled factors. It then remains difficult to determine which degradation principally caused an increase in localization error or a tracking failure. Even when one method achieves better overall performance, the result does not establish whether the improvement arises from effective handling of its target degradation. Moreover, existing datasets provide limited severity stratification within the same degradation factor. They therefore offer little evidence on how a SLAM system transitions from stable operation to degraded performance and eventual failure or on the conditions under which a method remains applicable. Degradation attribution and difficulty-trend analysis require evaluation data that treat one degradation factor as the primary controlled variable while keeping the underlying scene layout and other acquisition conditions as consistent as possible. Multiple severity conditions should then be established for that factor so that system performance can be related more clearly to both degradation source and severity.

Reliable six-degree-of-freedom (6-DoF) reference trajectories provide a second foundation for quantitative SLAM evaluation. Existing underwater datasets obtain reference information mainly through motion capture, acoustic positioning, or image-based offline reconstruction. In HAUD, for example, a motion-capture system observes an above-water marker assembly rigidly connected to the underwater camera and provides reference poses, but this approach requires a dedicated facility and multi-camera infrastructure [11,13]. Acoustic systems such as ultra-short baseline (USBL) positioning primarily provide position measurements and generally cannot independently recover a complete 6-DoF pose; their measurements can also be affected by sound-speed errors, installation offsets, and acoustic multipath [14]. Structure-from-motion (SfM) and COLMAP (The retained installation is version 3.6. https://colmap.github.io/ (accessed on 5 October 2026)) offer more flexible deployment, but trajectory reconstruction directly depends on underwater visibility, scene texture, and feature-matching quality [15]. Reconstruction completeness and reliability may consequently deteriorate under low illumination, motion blur, texture deficiency, or dynamic interference [16]. Quantitative evaluation under degraded conditions therefore also requires a 6-DoF reference-trajectory system that does not rely on dedicated multi-camera motion-capture infrastructure and whose estimates do not directly depend on the degraded underwater images.

To address these requirements, we introduce York-Tank, which is a controlled stereo dataset for underwater visual SLAM evaluation. York-Tank contains 16 test sequences collected in a shared tank scene and covers low illumination, non-uniform illumination, fast motion, texture deficiency, dynamic interference, and hybrid-degradation conditions. The dataset adopts a dominant-factor design: within each single-degradation sequence, one degradation factor is treated as the primary controlled variable while the underlying scene layout and other acquisition conditions are kept as consistent as possible. Based on the degradation type, condition severity, and factor combinations specified before acquisition, the sequences are organized into four levels: baseline, easy single degradation, hard single degradation, and hybrid degradation. Section 3 details the controlled settings and quantitative characterization. York-Tank is intended to complement datasets collected in real environments by providing repeatable controlled conditions for relating the SLAM performance to specific degradation factors and changes in difficulty.

For quantitative trajectory evaluation under degraded conditions, we further developed the York-Tank-GT reference-trajectory system. A single overhead camera observes an above-water AprilTag array rigidly connected to the underwater robot and estimates the array’s 6-DoF pose. A fixed rigid transformation between the array and the left camera then converts this pose into a left-camera reference trajectory. This design avoids placing artificial markers in the underwater visual scene, and reference-trajectory estimation does not directly rely on the degraded underwater stereo images. York-Tank provides 18,948 synchronized stereo image pairs acquired at 20 Hz with a total duration of approximately 15.8 min. A preview release containing the first baseline sequence is publicly available, and the complete dataset will be released in ROS (The retained processing workstation uses ROS 1 Noetic. https://github.com/ros/ros_comm (accessed on 5 October 2026)) bag and standalone-file formats after acceptance. The complete release will also provide camera calibration files, raw AprilTag-array trajectories, the fixed array-to-left-camera transformation, and evaluation tools.

The main contributions of this work are as follows:

(1) We introduce York-Tank, which is a controlled underwater stereo dataset that combines dominant-factor acquisition design with within-factor severity stratification. Documented acquisition settings and post-acquisition image and motion indicators characterize the recorded conditions, supporting targeted comparisons of SLAM performance across degradation types and relative severity levels. Additional hybrid sequences provide combined-degradation conditions.
(2) We develop York-Tank-GT to support trajectory evaluation under these degradation conditions. A single overhead camera observes an above-water AprilTag array rigidly connected to the underwater robot, providing left-camera reference trajectories without directly relying on degraded underwater stereo images or introducing artificial markers into the underwater evaluation scene.

## 2. Related Work

Existing underwater visual datasets target tasks including underwater navigation, visual SLAM, three-dimensional reconstruction, and long-term observation and consequently differ in acquisition environment, sequence organization, and reference-trajectory source. To clarify the design position of York-Tank, Table 1 compares representative underwater visual datasets in terms of acquisition setting, organization by dominant degradation factor, difficulty stratification, repeatability of controlled conditions, and reference-trajectory source.

Among datasets acquired under controlled conditions, HAUD and TANK use relatively stable tank environments to improve acquisition repeatability. HAUD organizes its test sequences primarily by motion trajectory and robot operating area without treating a specific visual degradation factor and its severity as explicit sequence-design variables [11]. TANK further defines easy, medium, and hard levels and is therefore one of the underwater SLAM evaluation datasets most closely related to York-Tank [12]. These levels, however, jointly vary motion speed, illumination, operating range, and visible structural features. Cross-level comparisons consequently represent aggregate performance under simultaneous changes in several acquisition conditions rather than performance as a single dominant degradation factor becomes more severe.

Datasets collected in real environments, including Caves, AQUALOC, and Eiffel Tower, place greater emphasis on scene realism and environmental diversity. Caves records low illumination, weak texture, suspended particles, and restricted visibility in underwater caves, whereas AQUALOC covers several real underwater environments [8,9]. Scene structure, platform motion, and imaging conditions are naturally coupled in such data. These datasets are therefore well suited to testing the overall performance in complex underwater environments but provide limited support for controlled comparisons centered on a specific dominant degradation factor. Eiffel Tower repeatedly observes the same deep-sea structure and primarily supports long-term three-dimensional reconstruction and temporal-change analysis rather than difficulty-stratified comparisons of SLAM methods [10]. Real-environment and controlled-tank datasets thus serve complementary purposes, but their sequences are generally not organized explicitly by a dominant degradation factor and its severity.

Existing datasets also use different methods to obtain reference trajectories for quantitative SLAM evaluation. Caves relies on artificial underwater landmarks and distance measurements; AQUALOC and Eiffel Tower mainly reconstruct trajectories offline from underwater images using SfM/COLMAP; HAUD uses an external motion-capture system; and TANK fuses underwater AprilTag observations with AQUA-SLAM odometry constraints [8,9,10,11,12]. Each approach has distinct operating requirements. Underwater landmarks and markers depend on their visibility and introduce artificial elements into the underwater scene. SfM/COLMAP trajectory reconstruction is affected by underwater image quality, whereas motion capture requires a dedicated facility and multi-camera infrastructure. York-Tank-GT instead uses an overhead camera to estimate the pose of an above-water AprilTag array rigidly connected to the underwater robot, after which a fixed rigid transformation generates the left-camera reference trajectory. This approach avoids artificial markers in the underwater visual scene, and its reference-trajectory estimates do not directly depend on the degraded underwater stereo images.

In summary, datasets collected in real environments and controlled tanks provide complementary foundations in scene realism, experimental repeatability, and quantitative trajectory evaluation. York-Tank is not intended to replace these resources. It instead combines dominant-factor sequence organization, within-factor severity stratification, and overhead-camera-based reference-trajectory acquisition in a controlled tank setting, thereby extending support for analyzing performance changes and failure trends under specific degradation conditions.

## 3. Dataset Design and Collection

This section describes the design and data-collection workflow of York-Tank. Test conditions were specified around dominant visual degradation factors and their severity under a shared tank scene and stereo-camera configuration. The following subsections describe the acquisition platform and sensor calibration, the York-Tank-GT reference-trajectory system, the underwater scene, sequence design and degradation quantification, and the released data formats.

### 3.1. Hardware Platform

The York-Tank acquisition platform comprised an onboard stereo imaging unit and the York-Tank-GT reference-trajectory unit. The onboard unit used a hardware-synchronized Arducam OV9281 stereo camera (Arducam Technology Co., Ltd., Hong Kong, China). Two VIOCEAN WL-1500 white-light underwater lamps (VIOCEAN, Qingdao, China) with independent pulse-width modulation (PWM) control were mounted on either side of the robot to establish different illumination conditions. Their manufacturer-rated power and luminous flux were 15 W and 1500 lm per lamp, respectively. Ambient illumination was provided by domestic incandescent lamps whose manufacturer and model could not be identified. York-Tank-GT used an Arducam OV2311 monocular camera (Arducam Technology Co., Ltd., Hong Kong, China) fixed above the tank and an above-water AprilTag array rigidly connected to the underwater robot [17]. Table 2 and Table 3 summarize the principal camera specifications.

**Figure 1:**
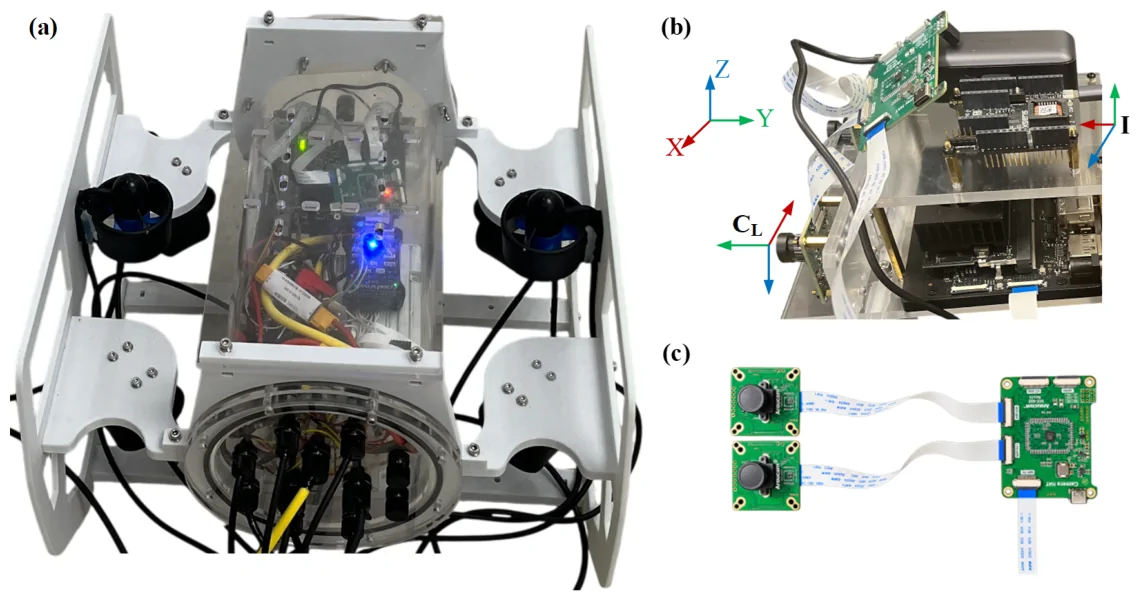
York-Tank acquisition platform and onboard stereo imaging configuration. (**a**) Underwater robot platform; (**b**) internal installation and coordinate frames of the onboard imaging and computing units; (**c**) stereo camera module.

Figure 1 shows the fixed hardware arrangement used throughout calibration and sequence collection. The stereo camera was mounted near the front viewport of the robot, and the two onboard lights were fixed on its sides. The overhead camera covered the operating region of the AprilTag array, which was connected to the underwater robot through a rigid support structure. These mounting relationships remained unchanged during underwater calibration and all sequence acquisitions, providing a common hardware configuration for stereo calibration, reference-trajectory generation, and cross-sequence comparison.

### 3.2. Sensor Calibration and Coordinate Frames

The calibration procedure established the geometric relationships among the onboard stereo images, overhead-camera observations, and left-camera reference trajectory. It comprised underwater stereo calibration, overhead monocular-camera calibration, and offline determination of the fixed rigid transformation between the AprilTag array and the left camera. The same calibration parameters were used for all test sequences because the cameras and AprilTag array remained fixed relative to their supporting structures during data collection.

(1) Underwater stereo-camera calibration: After installation inside the robot enclosure, synchronized left and right images of an independent AprilTag calibration board were recorded underwater from multiple viewpoints. The detected board corners and known board geometry were used to estimate the intrinsic parameters, distortion coefficients, and stereo extrinsics. These parameters were subsequently used for image undistortion and stereo rectification.
(2) Overhead monocular-camera calibration: The overhead camera was calibrated using the OpenCV (https://opencv.org/ (accessed on 5 October 2026). The retained processing workstation runs Python 3.8.10. Its current Python binding is OpenCV 4.13.0; the retained C++ SLAM binaries link to OpenCV 4.2.0) camera-calibration procedure [18,19]. Its intrinsic parameters and distortion coefficients were estimated from multi-view calibration-board images and were later used to estimate the AprilTag-array pose from corner observations.
(3) Determination of the fixed rigid transformation: The AprilTag array and left camera were connected through a rigid support structure. Their relative translation was derived from the nominal structural dimensions specified in the mechanical drawings, whereas their relative rotation was determined from the fixed mounting directions of the two coordinate frames. The resulting rigid transformation remained unchanged across all sequences and was used to convert array poses into left-camera reference poses. Assembly-induced uncertainty in this transformation has not been independently quantified.
(4) Coordinate frames and trajectory representation: The overhead-camera frame was used as the world frame *W*, the AprilTag-array frame was denoted by *B*, and the left-camera frame was denoted by $C_{L}$. The rigid transformation TYX maps coordinates from frame *Y* to frame *X*. The overhead camera directly estimated the raw array pose TBW(t). Given the fixed transformation TCLB from the left-camera frame to the array frame, the left-camera reference pose was computed as
  (1) TCLW(t)=TBW(t)TCLB.

The dataset provides the raw AprilTag-array trajectory TBW(t) and fixed transformation TCLB. The accompanying scripts use these quantities to generate the left-camera reference trajectory TCLW(t).

### 3.3. York-Tank-GT Reference-Trajectory Estimation

**Figure 2:**
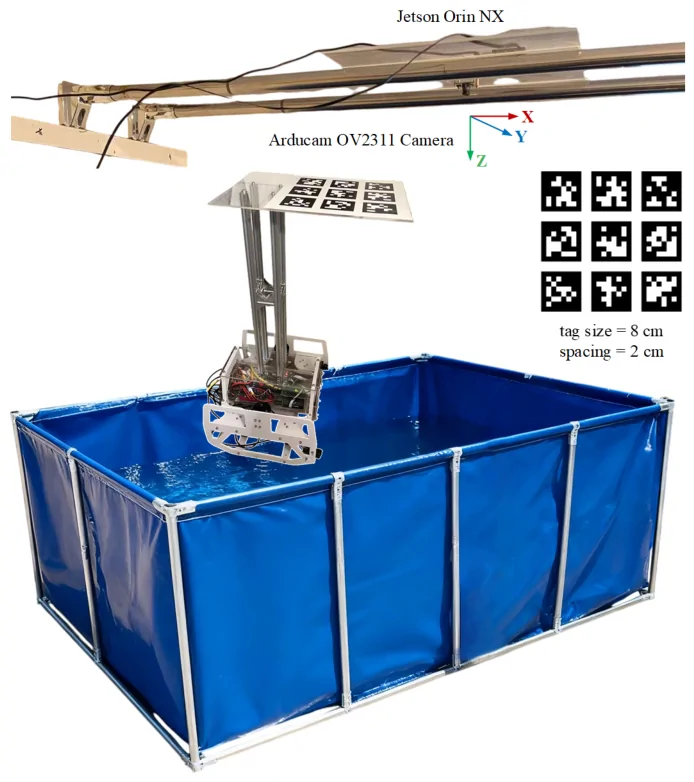
Overview of the York-Tank-GT setup.

York-Tank-GT was designed to provide 6-DoF reference trajectories for quantitative SLAM evaluation under severe underwater image degradation. As shown in Figure 2, the system comprised a monocular camera fixed above the tank and an above-water AprilTag array rigidly connected to the underwater robot. The overhead camera directly estimated the array pose in the world frame. The fixed transformation defined in Section 3.2 then converted this pose into a left-camera reference pose. This design avoided placing artificial markers in the underwater visual scene, and trajectory estimation did not directly depend on the degraded onboard stereo images.

The camera field of view, tag layout, and mechanical connection were designed jointly to maintain stable array observations. The overhead camera covered the complete operating region of the array. The array used a 3×3 grid of tags, which each had an 8 cm side length and 2 cm spacing between adjacent tags. Multiple tags provided redundant corner observations when individual tags were occluded or missed. Four rigid columns connected the array to the underwater robot, and four auxiliary supports near the center limited relative motion and structural deformation.

For each overhead-camera frame, visible AprilTags were detected and their corner coordinates were extracted. The known array geometry established the corresponding 2D–3D point pairs. Iterative perspective-n-point (PnP) estimation was applied to the first five frames of each sequence to initialize the trajectory and establish the preceding-pose reference used for temporal continuity. After initialization, the infinitesimal plane-based pose estimation (IPPE) solver generated additional pose candidates, which were refined by iterative optimization [20].

To reduce orientation jumps caused by switching between planar-PnP solutions, candidate selection considered both corner reprojection error and motion relative to the preceding pose. For candidate *k*, let $E_{k}$ denote the reprojection error, $\Delta p_{k}$ the translation change, and $\Delta \theta_{k}$ the rotation change. Its score was defined as

(2) $$J_{k}=E_{k}+60\;\Delta p_{k}+0.2\;\Delta \theta_{k}.$$

Here, $E_{k}$ is measured in pixels, $\Delta p_{k}$ in meters, and $\Delta \theta_{k}$ in degrees. The same weights were used for all test sequences, and the candidate with the lowest score was selected for each frame. This criterion combined image-space geometric consistency with inter-frame motion continuity to reduce the discontinuities caused by candidate switching.

**Figure 3:**
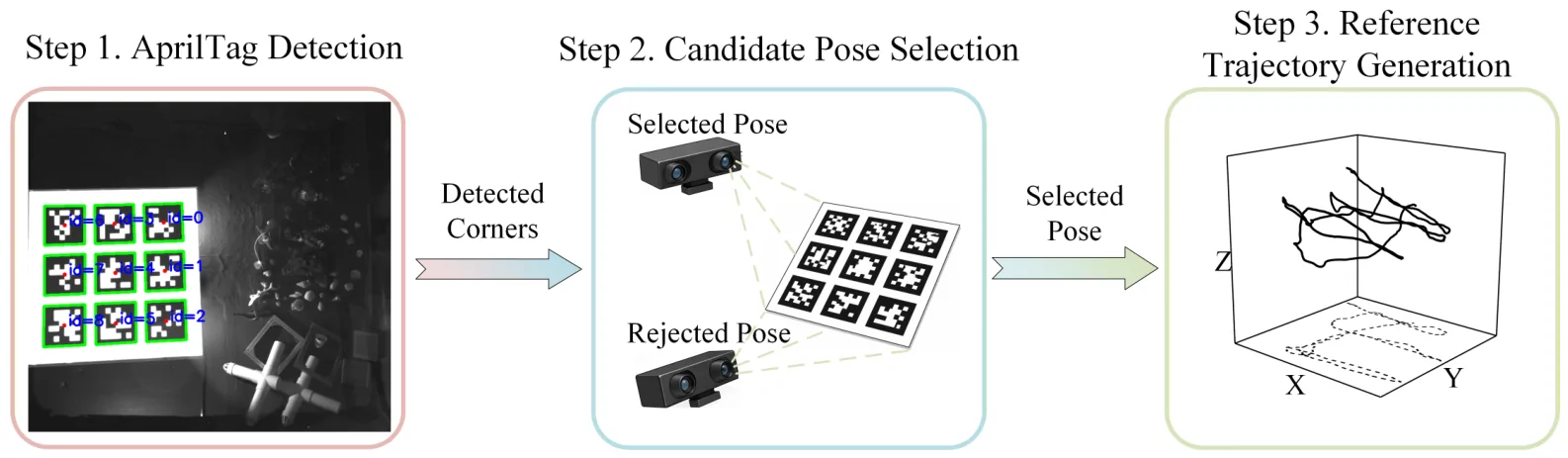
Pipeline for York-Tank-GT reference-trajectory estimation. Colored panel outlines distinguish the three processing stages. In the first panel, green outlines mark detected tags, red markers indicate tag locations, and blue labels give tag identifiers. Dashed green lines in the second panel schematically illustrate candidate viewing geometry.

After obtaining the raw array poses, the fixed rigid transformation in Section 3.2 was applied to generate left-camera reference poses. The timestamped poses formed a 6-DoF left-camera reference trajectory, as summarized in Figure 3.

The onboard stereo device and York-Tank-GT device synchronized their system clocks using Chrony (The retained Jetson installation is version 3.5. https://chrony-project.org/ (accessed on 5 October 2026)) before data collection. Each device then recorded image timestamps using the synchronized system clock, providing a common temporal basis for the stereo images and reference trajectory.

### 3.4. Underwater Scene

**Figure 4:**
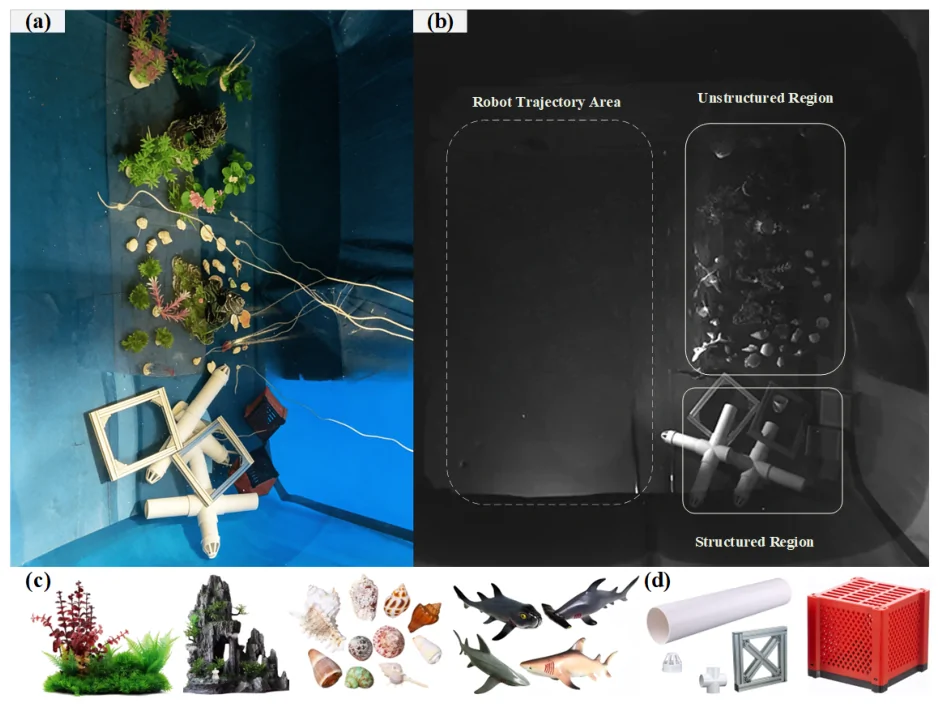
York-Tank underwater scene. (**a**) Tank environment; (**b**) scene layout, including the robot operating area, natural-element area, and engineered-structure area; (**c**) representative objects in the natural-element area; (**d**) representative objects in the engineered-structure area.

York-Tank was collected in a tank measuring 1.5×1.5×1 m. As shown in Figure 4, the scene contained a robot operating area, a natural-element area, and an engineered-structure area. Unoccupied tank walls served as texture-deficient viewing regions. Together, these areas provided visual content with different texture densities and geometric structures within one shared scene.

The natural-element area contained aquatic plants, rocks, shells, and artificial animal models with irregular geometry and non-uniform texture. The engineered-structure area contained pipes, frames, and regular geometric objects that provided distinct edges, planes, and structural patterns. These areas and the texture-deficient tank walls formed the base scene used for all degradation sequences.

The base scene layout remained unchanged across sequences. Only the illumination, robot motion, primary viewing region, and dynamic-object configuration were adjusted according to the preset degradation condition. This design reduced the scene-structure variation between sequences and provided a consistent basis for evaluating different degradation conditions.

### 3.5. Sequence Design, Degradation Settings, and Quantitative Characterization

York-Tank was designed along two dimensions, dominant degradation factor and difficulty level, under the fixed base scene described in Section 3.4. Before data collection, four difficulty levels were defined for illumination degradation, fast motion, texture deficiency, and dynamic interference. These levels comprised baseline, easy single degradation, hard single degradation, and hybrid degradation, and they were used to organize 16 test sequences. Each condition was established through preset illumination parameters, robot motion, primary viewing region, and dynamic-object configuration. After collection, image statistics and reference trajectories were used to quantify the resulting degradations and verify that the preset conditions produced the intended differences.

#### 3.5.1. Sequence Organization by Dominant Degradation Factor

A dominant degradation factor was defined as the acquisition variable deliberately adjusted within a group of controlled sequences and treated as the primary manipulated condition in within-group comparisons. Two baseline sequences provided a common reference for all single-degradation conditions. For each degradation form, the easy and hard sequences retained the same degradation type while changing its preset severity. Hybrid-degradation sequences were not used for within-factor severity comparisons; instead, they assessed system performance when several degradation factors were combined. Because complete physical decoupling is impractical, the design emphasized one primary variable while keeping other acquisition conditions as consistent as possible.

#### 3.5.2. Preset Degradation Conditions and Acquisition Control

The two baseline sequences served as the common reference, and easy and hard conditions were specified for each single-degradation form. The stereo camera used the same exposure time and gain in all sequences with the gain fixed at zero. Under the baseline condition, the PWM commands of both onboard lights were 1600. Low illumination and non-uniform illumination were generated by adjusting the two PWM commands. Fast motion was established by changing the translational speed of the robot, whereas texture deficiency was controlled through the primary viewing region. Dynamic interference was introduced by moving a toy shark attached to a wooden rod within the stereo-camera field of view. The easy and hard dynamic conditions differed in the image area occupied by the moving object. Table 4 lists the detailed settings.

The table lists only the baseline, easy, and hard single-degradation conditions. Level 4 sequences combined two or more preset conditions, as detailed in Section 3.5.4. Section 3.5.3 quantifies the degradation characteristics and severity that were actually produced after acquisition.

**Figure 5:**
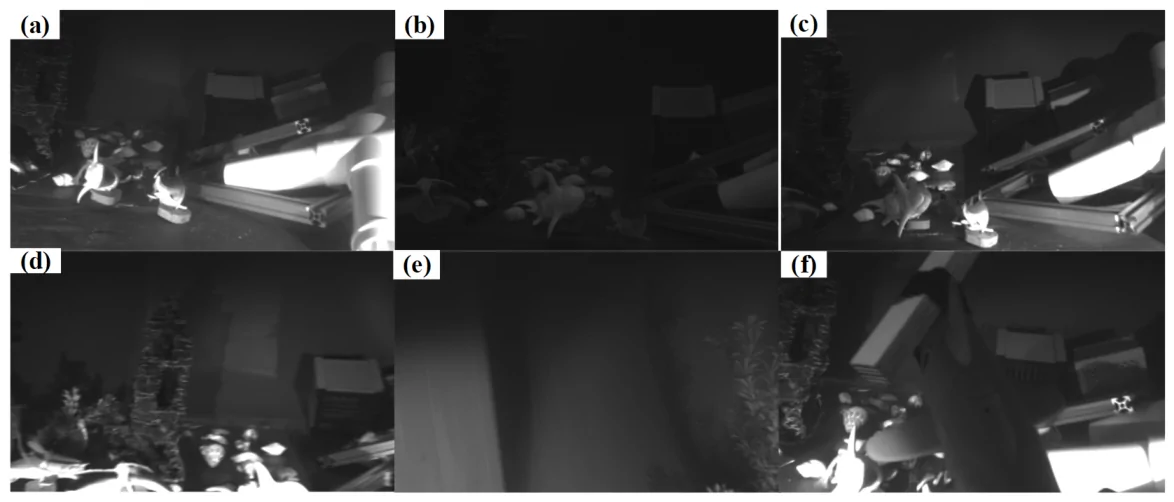
Representative images of the baseline and five preset degradation forms under the shared scene layout. (**a**) Baseline; (**b**) low illumination; (**c**) non-uniform illumination; (**d**) fast motion; (**e**) texture deficiency; (**f**) dynamic interference.

Figure 5 shows representative images of the five preset degradation forms under the shared base scene layout.

#### 3.5.3. Degradation Quantification and Verification of Preset Conditions

Post-acquisition indicators were used to verify whether the easy and hard settings in Table 4 produced the intended relative severity. Separate image or motion indicators were selected for low illumination, non-uniform illumination, fast motion, texture deficiency, and dynamic interference. These indicators characterized and verified the preset conditions; they were not used to redefine the difficulty levels. Level 4 was excluded from within-factor progression because it combined multiple degradation factors.

The image indicators for low illumination, non-uniform illumination, and texture deficiency were calculated from all original-resolution left-camera grayscale images. Let $I_{t}\left(x\right)$ denote the grayscale value at pixel *x* in frame *t*, *N* the number of pixels in each frame, and $Q_{p}\left(\cdot \right)$ the *p*th percentile.

Low illumination was characterized by the median frame-mean intensity *B*:

(3) $$B={\mathrm{median}}_{t}\;\left[\frac{1}{N}\sum_{x}I_{t}\left(x\right)\right].$$

Non-uniform illumination was characterized by the spatial intensity range *U* of a low-frequency illumination field. For frame *t*, Gaussian filtering suppressed local texture and edges to obtain $L_{t}$. The difference between the 90th and 10th percentiles was then calculated, and the median across all frames was used:

(4) $$L_{t}=G_{\sigma }*I_{t},$$

(5) $$U={\mathrm{median}}_{t}\;\left[Q_{90}\left(L_{t}\right)-Q_{10}\left(L_{t}\right)\right].$$

Here, $G_{\sigma }$ is a Gaussian kernel with standard deviation σ, and ∗ denotes convolution. We set σ=W/24, where *W* is the image width. For the original width of 1280 pixels, σ=53.3 pixels. A larger *U* indicates a stronger spatial illumination difference.

Fast motion was characterized by the 90th percentile of linear speed, $V_{90}$, which was calculated from the York-Tank-GT reference trajectory. Let $p_{i}$ and $p_{i+1}$ be positions at adjacent timestamps $\tau_{i}$ and $\tau_{i+1}$. The instantaneous speed and sequence-level indicator were defined as

(6) $$v_{i}=\frac{{∥p_{i+1}-p_{i}∥}_{2}}{\tau_{i+1}-\tau_{i}},$$

(7) $$V_{90}=Q_{90}\;\left(\{v_{i}\}\right).$$

A larger $V_{90}$ indicates more pronounced high-speed translation. The 90th percentile was used instead of the maximum to reduce sensitivity to isolated speed outliers.

Texture deficiency was characterized by the median brightness-normalized gradient $T_{n}$, which reduced the effect of overall image brightness on gradient magnitude:

(8) $$T_{n}={\mathrm{median}}_{t}\;\left[\frac{\frac{1}{N}\sum_{x}\sqrt{{\left(K_{x}*I_{t}\left(x\right)\right)}^{2}+{\left(K_{y}*I_{t}\left(x\right)\right)}^{2}}}{\frac{1}{N}\sum_{x}I_{t}\left(x\right)+\varepsilon }\right].$$

Here, $K_{x}$ and $K_{y}$ are the horizontal and vertical 3×3 Sobel operators, and $\varepsilon =10^{-6}$ prevents division by zero. A smaller $T_{n}$ indicates weaker image gradients relative to overall brightness and therefore fewer texture and edge structures.

Dynamic regions were extracted using the nano segmentation variant of You Only Look Once version 8 (YOLOv8n-seg), which was implemented with the Ultralytics framework (version 8.4.41 for model training) [21,22]. The model was trained to segment the toy shark and its supporting rod. The training image-size parameter was set to 640, and a confidence threshold of 0.25 was used during evaluation. Let $M_{t}\left(x\right)$ be the union mask of the two target classes in frame *t*. The 95th percentile of dynamic-region area was defined as

(9) $$d_{t}=\frac{1}{N}\sum_{x}M_{t}\left(x\right),$$

(10) $$D_{95}=100\times Q_{95}\;\left(\{d_{t}\}\right).$$

Frames in which no dynamic object was detected contributed a value of zero. Table 5 reports the resulting indicators for the single-degradation conditions.

For Level 1, each value in Table 5 is the mean of the two sequence-level indicators from the baseline sequences. The Level 2 and Level 3 values were obtained from the corresponding easy and hard sequences. From Level 1 to Level 3, *B* and $T_{n}$ decreased, whereas *U*, $V_{90}$, and $D_{95}$ increased. These changes confirm that the acquired easy and hard sequences produced relative degradation differences consistent with the preset conditions.

#### 3.5.4. Sequence Composition and Dataset Scale

York-Tank contains 16 test sequences: two Level 1 baseline sequences, ten single-degradation sequences forming five Level 2–Level 3 pairs, and four Level 4 hybrid-degradation sequences. The single-degradation sequences cover low illumination, non-uniform illumination, fast motion, texture deficiency, and dynamic interference with one easy and one hard sequence for each form. Hybrid-degradation sequences combine two or more of these conditions. Table 6 lists the degradation composition, difficulty level, number of stereo pairs, and duration of each sequence.

The 16 sequences comprise 18,948 synchronized stereo pairs with a total duration of 946.2 s or approximately 15.8 min. Individual sequence durations range from 45.0 to 72.5 s.

### 3.6. Data Organization and Formats

York-Tank is organized in Robot Operating System (ROS) bag and standalone-file formats. A preview release containing Sequence 1 is publicly available on Zenodo at https://doi.org/10.5281/zenodo.20432375. The complete 16-sequence dataset will be released after acceptance. ROS bags support stereo-image playback and direct use with SLAM systems in ROS, whereas the standalone format supports offline processing and non-ROS frameworks. Each sequence in the complete release will provide synchronized rectified left and right images and the raw AprilTag-array trajectory. The fixed transformation from the array frame to the left-camera frame will also be provided together with scripts that generate the left-camera 6-DoF reference trajectory. The complete release will further include camera calibration, sequence metadata, and trajectory-evaluation tools.

#### 3.6.1. ROS Bag Format

Each test sequence will be provided as an ROS bag containing the left and right image streams rectified using the underwater calibration parameters. There are two image topics:

(1) /camera/left/image_rect_underwater (sensor_msgs/Image): rectified underwater left-camera images.
(2) /camera/right/image_rect_underwater (sensor_msgs/Image): rectified underwater right-camera images.

Both image streams retain the timestamps recorded during synchronized acquisition and can be replayed directly as input to a stereo visual SLAM system in ROS.

#### 3.6.2. Standalone-File Format

For offline and non-ROS use, each sequence will also be provided as timestamp-named left and right images and a text file containing the raw AprilTag-array trajectory. Each trajectory entry contains a timestamp, position, and orientation. The accompanying script uses the fixed array-to-left-camera transformation to generate the corresponding left-camera 6-DoF reference trajectory.

#### 3.6.3. Calibration Files and Evaluation Tools

Calibration results will be provided as YAML files containing camera intrinsics, distortion parameters, and stereo extrinsics for SLAM configuration. The complete release will also include sequence metadata and trajectory-evaluation scripts for generating the left-camera reference trajectory, associating estimated and reference trajectories, and calculating localization errors.

## 4. SLAM Evaluation Results and Discussion

This section evaluates York-Tank under a unified input and evaluation protocol. We first compare trajectory completeness and the root mean square error (RMSE) of translational absolute trajectory error (ATE) for ORB-SLAM3 (The retained source trees are based on commit 6c3b525, with local modifications. https://github.com/UZ-SLAMLab/ORB_SLAM3 (accessed on 5 October 2026)) and SVO Pro (The retained source tree is based on commit ca371f3, with local modifications. https://github.com/uzh-rpg/rpg_svo_pro_open (accessed on 5 October 2026)) across all 16 sequences. We then use ORB-SLAM3 as a fixed baseline and separately add contrast-limited adaptive histogram equalization (CLAHE) and YOLO-based dynamic-region feature rejection. These controlled front-end comparisons relate performance changes to the target degradation and examine how the operating range and observed failure trend of each intervention change with degradation severity. The results of both SLAM systems are subsequently aggregated across Levels 1–4 to test whether the preset difficulty stratification produces progressive performance changes. Finally, York-Tank-GT is evaluated using AprilTag observations, PnP reprojection error, trajectory-output continuity, and consistency with trajectories reconstructed by COLMAP.

### 4.1. Evaluation Methods and Experimental Design

The experiments use the controlled tank sequences described in Section 3.5 with acquisition settings and measured degradation indicators reported in Table 4 and Table 5, respectively. The cross-system comparison examines system-dependent responses to the same recorded sequences. The controlled front-end comparisons change only the selected front-end processing within ORB-SLAM3. Comparisons across target and non-target degradations assess whether the observed effects depend on the degradation condition, while easy–hard comparisons examine how these effects vary with severity. Hybrid sequences assess the overall response under combined degradations and are not used for single-factor attribution.

The cross-system comparison uses ORB-SLAM3 [23] and the open-source SVO Pro implementation [24,25]. Both accept stereo images and output 6-DoF trajectories, allowing evaluation under the same input and scoring protocol. ORB-SLAM3 is a feature-based full SLAM system, whereas SVO Pro uses a semi-direct visual-estimation framework. Their distinct image-processing and pose-estimation mechanisms enable the same sequences to reveal system-specific sensitivity to particular degradation conditions.

The controlled front-end comparisons use ORB-SLAM3, a representative feature-based system, as a fixed baseline. CLAHE [26] is applied at the image-input stage as an intervention targeting low illumination. A clip limit of 2.0 and a tile grid size of 8×8 are used as a fixed configuration without additional parameter tuning.

For dynamic-region feature rejection, a YOLOv8n-seg model [21,22] was trained using approximately one thousand manually annotated images from separately collected dynamic-object sequences. The annotations covered the toy shark and its supporting rod. Training and evaluation were separated at the sequence level: no images from the released evaluation sequences were used for training. The training image-size parameter was set to 640 to reduce training computational cost. A confidence threshold of 0.25 was adopted based on visual inspection of the segmentation outputs. During evaluation, the left and right images and their corresponding dynamic-region masks were provided to the SLAM front end, where feature points within the masked regions were rejected. All other ORB-SLAM3 procedures and parameters remain unchanged.

All methods and configurations are evaluated on the same 16 York-Tank sequences using identical stereo images, camera calibration parameters, York-Tank-GT reference trajectories, and evaluation code. Each method retains one configuration across all sequences. Section 4.2 defines the evaluation metrics and trajectory-processing procedure.

### 4.2. Evaluation Protocol and Metrics

All estimated trajectories are processed using a common procedure for temporal association, trajectory segmentation, coordinate-frame alignment, and error calculation. For each sequence, we first determine the temporal interval jointly covered by the left-camera images and the left-camera reference trajectory generated by York-Tank-GT. Within this interval, each reference pose is associated with its temporally nearest estimated pose. The maximum permitted timestamp difference is 0.10 s, and each estimated pose can be used in at most one association.

Tracking loss or anomalous pose jumps can divide an estimated trajectory into discontinuous segments. Consecutive estimated poses are therefore assigned to different segments when their timestamp interval exceeds 2 s or when the Euclidean distance between their positions exceeds 0.15 m. After temporal association, segments containing fewer than 50 valid pose pairs are excluded. Among the remaining segments, the segment with the largest number of valid pose pairs is selected as the longest continuous valid trajectory segment.

SLAM performance is evaluated using trajectory completeness and translational ATE RMSE. Let $N_{I}$ be the number of left-camera frames in the common evaluation interval and $N_{V}$ the number of successfully associated pose pairs in the longest continuous valid trajectory segment. Trajectory completeness *C* is defined as

(11) $$C=\frac{N_{V}}{N_{I}}\times 100\%.$$

Trajectory completeness measures the ability of a system to maintain continuous tracking over the complete evaluation interval. A higher value indicates that the system outputs valid poses over a larger proportion of that interval.

Within the longest continuous valid segment, the estimated trajectory is aligned with York-Tank-GT using an SE(3) rigid transformation without scale correction. Let $p_{i}^{\mathrm{gt}}$ and $p_{i}^{\mathrm{est}}$ denote the reference and estimated positions for the *i*th associated pose pair, respectively, and let R and t denote the rotation matrix and translation vector obtained through alignment. The translational error is

(12) $$e_{i}={∥p_{i}^{\mathrm{gt}}-\left(Rp_{i}^{\mathrm{est}}+t\right)∥}_{2}.$$

The translational ATE RMSE is then defined as

(13) $${\mathrm{ATE}}_{\mathrm{RMSE}}=\sqrt{\frac{1}{N_{\mathrm{seg}}}\sum_{i=1}^{N_{\mathrm{seg}}}e_{i}^{2}}.$$

Here, $N_{\mathrm{seg}}$ is the number of associated pose pairs in the selected segment.

ATE RMSE is reported in meters and characterizes localization accuracy within the longest continuous valid trajectory segment. Trajectory completeness and ATE RMSE respectively describe continuous tracking ability and localization accuracy over the valid tracking interval, so they are interpreted jointly. ATE RMSE is reported whenever a result contains a valid segment that supports temporal association and trajectory alignment. A result is marked N/A only when the system produces no valid trajectory segment.

### 4.3. Per-Sequence Evaluation Results

We first compare ORB-SLAM3 and SVO Pro across all 16 sequences to determine whether York-Tank reveals performance differences between visual-estimation mechanisms under a common evaluation protocol. Table 7 reports trajectory completeness and ATE RMSE.

On the two baseline sequences, trajectory completeness is 99.76–99.85% for ORB-SLAM3 and 95.74–96.52% for SVO Pro, while ATE RMSE lies between 0.0411 and 0.0487 m for both systems. These results provide a common baseline for comparison with the degraded sequences. The systems respond differently once degradation is introduced. On both fast-motion sequences, ORB-SLAM3 maintains nearly 100% completeness, whereas SVO Pro reaches only 56.09% and 54.06%. On the hard dynamic-interference sequence, SVO Pro achieves higher completeness but also a larger ATE RMSE than ORB-SLAM3. These results demonstrate algorithm-dependent sensitivity to the recorded degradations. The subsequent findings for CLAHE and dynamic-region feature rejection are limited to the evaluated ORB-SLAM3 configurations.

We next hold ORB-SLAM3 fixed and separately add CLAHE and YOLO-based feature rejection in dynamic regions. Table 8 reports the results of all three configurations across the complete test set.

### 4.4. Degradation Attribution and Method Operating Ranges

To relate the performance changes caused by each front-end intervention to its target degradation, the per-sequence results in Table 8 are grouped by dominant degradation condition. We then calculate the mean change of each front-end configuration relative to the ORB-SLAM3 baseline. Let $C_{b}$ and $C_{m}$ denote the mean trajectory completeness of the baseline and modified configurations, respectively, within a degradation group. The change in trajectory completeness is defined as

(14) $$\Delta C=C_{m}-C_{b}.$$

Let $E_{b}$ and $E_{m}$ denote the corresponding mean ATE RMSE values. Their relative change is defined as

(15) $$\Delta E=\frac{E_{m}-E_{b}}{E_{b}}\times 100\%.$$

Here, ΔC is measured in percentage points (pp), and ΔE<0 indicates reduced localization error. For each single-degradation group, the completeness change is calculated jointly from its easy and hard sequences. The ATE RMSE change includes only sequences for which both the baseline and the corresponding modified configuration produce valid trajectories. Table 9 reports the results.

CLAHE has its largest effect on the low-illumination sequences. On the easy low-illumination sequence, it increases trajectory completeness from 61.26% to 88.82%, while ATE RMSE increases from 0.027366 to 0.033279 m. The higher completeness indicates that CLAHE provides continuous valid pose estimates over a larger proportion of the evaluation interval. However, ATE RMSE is calculated over each configuration’s own longest continuous valid trajectory segment, which may cover different time intervals. The reported ATE values therefore do not establish whether localization accuracy improves or worsens over a common tracking interval. On the hard low-illumination sequence, the ORB-SLAM3 baseline produces no valid trajectory, whereas the CLAHE configuration yields a trajectory with 76.38% completeness and an ATE RMSE of 0.029591 m. Because the baseline fails completely on this sequence, an ATE RMSE change cannot be calculated. The completeness result nevertheless shows that CLAHE changes the outcome from tracking failure to sustained valid pose output.

The YOLO configuration has its largest effect on the dynamic-interference sequences. On the easy dynamic-interference sequence, trajectory completeness changes only from 98.09% to 99.74%, while ATE RMSE decreases by 20.49%, from 0.073617 to 0.058532 m. Dynamic-region feature rejection therefore acts primarily by reducing localization error on this sequence. On the hard dynamic-interference sequence, completeness changes from 53.53% to 53.44%, and ATE RMSE changes from 0.056703 to 0.056484 m. Both differences are small, indicating that the improvement observed under the easy condition does not persist under hard dynamic interference.

**Figure 6:**
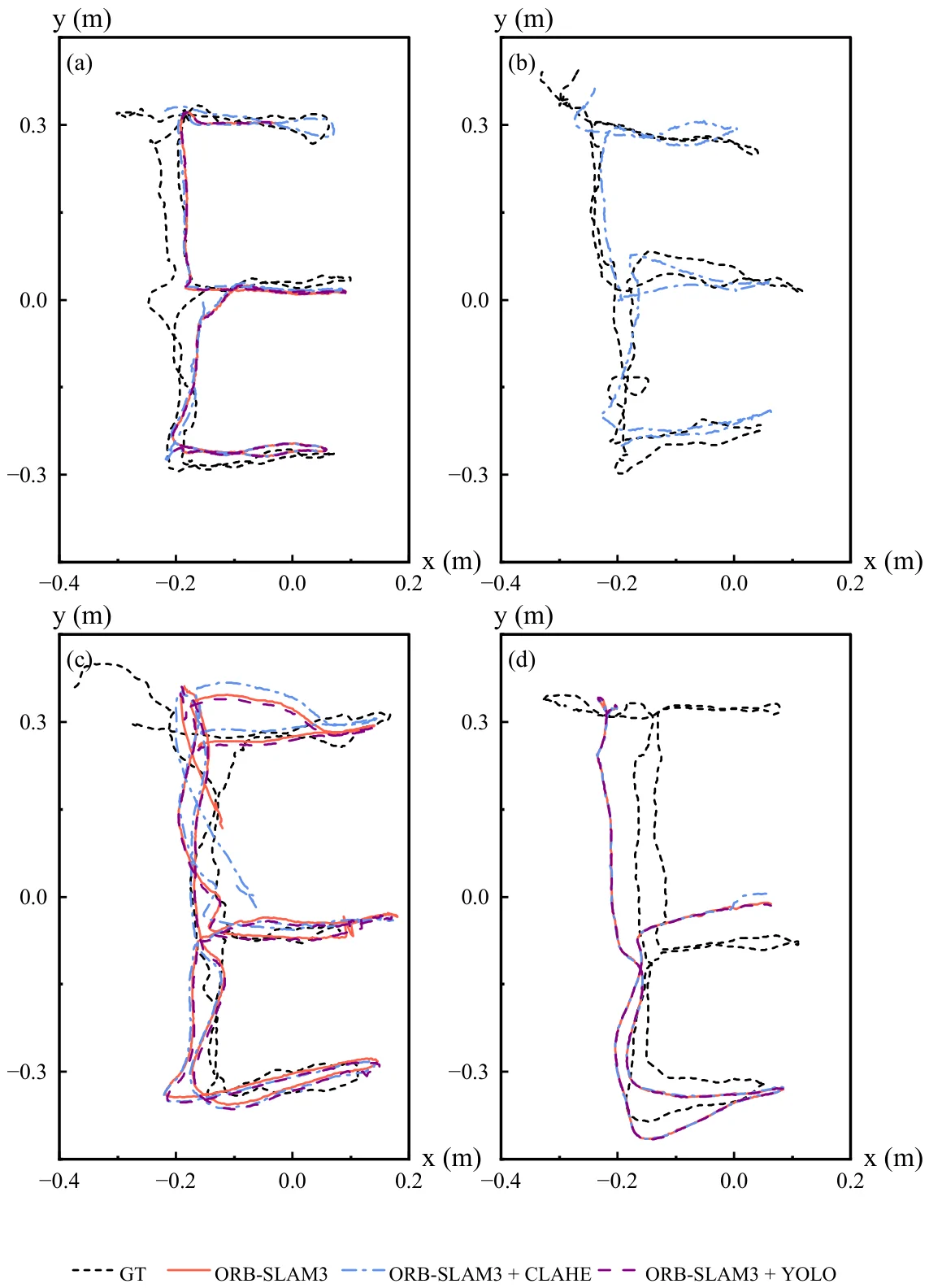
Representative trajectories of ORB-SLAM3 and the corresponding front-end configurations under low illumination and dynamic interference: (**a**) 3_dark_easy; (**b**) 4_dark_hard; (**c**) 11_dynamic_easy; and (**d**) 12_dynamic_hard.

Figure 6 provides representative trajectory comparisons corresponding to these quantitative results. CLAHE produces a longer continuous valid trajectory on the low-illumination sequences. On the easy dynamic-interference sequence, the YOLO configuration reduces the deviation between the estimated and reference trajectories, whereas the hard sequence does not show a comparable improvement.

Under non-target degradations, including non-uniform illumination, fast motion, and texture deficiency, both front-end configurations have generally small effects on trajectory completeness and ATE RMSE and produce no consistent improvement. On the hybrid-degradation sequences, CLAHE and YOLO increase mean completeness by 12.67 and 9.24 percentage points, respectively, but the direction and magnitude of change vary across individual sequences. Because each hybrid sequence contains several degradation factors, these results characterize the overall response of each intervention under compound conditions and are not used to attribute performance to a single factor.

Together, Table 8 and Table 9 and Figure 6 show that the largest effects of CLAHE and YOLO occur on the low-illumination and dynamic-interference sequences, respectively, with no consistent effect across most non-target conditions. York-Tank also distinguishes whether an intervention improves continuous tracking or localization accuracy within valid trajectory segments and reveals how these effects change as degradation becomes more severe. It therefore provides controlled evidence for analyzing the operating ranges and observed failure trends of degradation-targeted methods.

### 4.5. Validation of the Difficulty Stratification

As described in Section 3, the 16 sequences are organized into Levels 1–4 according to the degradation type and severity specified before acquisition. These levels correspond to baseline, easy single degradation, hard single degradation, and hybrid degradation, respectively. This subsection aggregates the ORB-SLAM3 and SVO Pro results to examine whether the four levels produce progressive performance changes across different visual-estimation systems. Levels 1–4 contain 2, 5, 5, and 4 sequences, respectively. When calculating mean trajectory completeness, sequences without a valid trajectory are assigned a completeness of zero. Mean ATE RMSE is calculated only from sequences with valid trajectories. Table 10 presents the results.

Mean trajectory completeness decreases monotonically for both systems across Levels 1–4. For ORB-SLAM3, it falls from 99.81% to 83.17%, 58.82%, and 41.36%; for SVO Pro, it falls from 96.13% to 56.94%, 49.89%, and 39.89%. Although both systems preserve the same ordering across difficulty levels, their decline differs. SVO Pro shows a pronounced completeness reduction between Levels 1 and 2, whereas ORB-SLAM3 retains relatively high completeness at Level 2 before declining further at Levels 3 and 4.

Among sequences for which valid trajectories are obtained, the mean ATE RMSE also increases monotonically with difficulty. It rises from 0.043504 to 0.093870 m for ORB-SLAM3 and from 0.045442 to 0.081172 m for SVO Pro. Because mean ATE RMSE excludes sequences without valid trajectories, it must be interpreted together with completeness. As difficulty increases, both systems maintain continuous tracking over a smaller proportion of each sequence, while localization error within the remaining valid segments generally increases.

ORB-SLAM3 and SVO Pro therefore both progress from high-completeness, low-error performance toward low-completeness, high-error performance while retaining their system-specific degradation sensitivities. The four preset levels do not merely reflect the behavior of one SLAM system. They produce a consistent progression from baseline to more severely degraded conditions across distinct visual-estimation mechanisms and support the analysis of the transition from stable tracking to partial tracking and failure.

### 4.6. Observation Consistency and Trajectory Usability of York-Tank-GT

York-Tank-GT is evaluated from two complementary perspectives: the internal observation consistency of the system and its trajectory consistency with independent visual reconstruction. First, we calculate the proportion of valid PnP poses, the number of visible AprilTags per frame, and the reprojection error across all 16 sequences. These quantities characterize the continuous output of the raw array trajectory and its geometric consistency with observations from the overhead camera. We then compare the left-camera reference trajectory generated from the raw array trajectory with COLMAP reconstructions on representative sequences. COLMAP serves here as an independent visual-reconstruction reference—not as a higher-accuracy reference trajectory.

To match the data used in the SLAM evaluation, all statistics are calculated only over the common acquisition interval of each stereo sequence and York-Tank-GT. Let $N_{t}$ be the number of detected tag corners in frame *t*, $u_{t,j}$ the observed location of corner *j*, and ${\hat{u}}_{t,j}$ its position reprojected using the PnP pose. The frame-wise reprojection RMSE is defined as

(16) $$e_{t}=\sqrt{\frac{1}{N_{t}}\sum_{j=1}^{N_{t}}{∥u_{t,j}-{\hat{u}}_{t,j}∥}_{2}^{2}}.$$

The proportion of valid PnP poses evaluates the continuity of the raw array-trajectory output, the mean number of visible tags per frame characterizes the available array observations, and the 95th percentile of frame-wise reprojection RMSE characterizes the geometric consistency between the PnP poses and observed image corners. Table 11 reports these statistics.

Every overhead-camera frame within the common acquisition intervals of the 16 sequences produces a valid PnP pose. On average, 8.90 AprilTags are visible per frame, and 95% of frames have a reprojection RMSE no greater than 1.102 px, indicating sustained array visibility and stable geometric fitting during acquisition. The minimum tag visibility occurs in 17 frames of 10_fewtexture_hard, in which only three tags are detected per frame. Valid poses are nevertheless obtained continuously in these frames with a maximum reprojection RMSE of 0.323 px. The remaining tag corners therefore continue to provide usable constraints for PnP pose estimation when part of the array is temporarily not visible.

To further assess the consistency between York-Tank-GT and a separately reconstructed visual trajectory, representative sequences spanning Levels 1–4 are reconstructed offline from the underwater stereo images using COLMAP [15]. Before comparison, the fixed array-to-left-camera transformation converts the raw AprilTag-array trajectory into a left-camera reference trajectory. Timestamp association is then established between this trajectory and the COLMAP trajectory, which is followed by scale and coordinate-frame alignment using a Sim(3) transformation. COLMAP reconstruction completeness is defined as the proportion of sequence images that are successfully registered and assigned a pose. The deviation between the two trajectories is measured using aligned ATE RMSE. Table 12 presents the comparison.

COLMAP registers all images in the representative Level 1 and Level 2 sequences and produces trajectories with relatively small deviations from the York-Tank-GT left-camera references. It still completes reconstruction on the Level 3 sequence, although the deviation between the trajectories increases. On the two Level 4 hybrid-degradation sequences, COLMAP, respectively, produces only a partial reconstruction or no valid trajectory. York-Tank-GT, by contrast, maintains complete trajectory output on all representative sequences.

Taken together, the AprilTag observations, PnP reprojection errors, and COLMAP comparisons show that York-Tank-GT continuously generates raw array trajectories throughout the test set and converts them into left-camera reference trajectories using the fixed transformation. Its output remains continuous when COLMAP reconstruction based on degraded underwater images becomes incomplete or fails, supporting quantitative underwater visual SLAM evaluation across the degradation conditions represented in York-Tank.

## 5. Conclusions

This work presents York-Tank, which is a controlled dataset for underwater stereo visual SLAM evaluation. The dataset contains 16 test sequences and 18,948 synchronized stereo image pairs. Under a shared underlying scene layout and camera configuration, its test conditions are organized around illumination degradation, fast motion, texture deficiency, and dynamic interference with four difficulty levels comprising baseline, easy single degradation, hard single degradation, and hybrid degradation. The complete release will include camera calibration files and evaluation tools together with raw AprilTag-array trajectories generated by York-Tank-GT and the fixed array-to-left-camera transformation required to obtain 6-DoF left-camera reference trajectories for SLAM evaluation.

Cross-system evaluation shows that the mean trajectory completeness of both ORB-SLAM3 and SVO Pro decreases monotonically with increasing difficulty, while ATE RMSE within valid trajectory segments generally increases. The preset levels therefore produce progressive test conditions across different visual-estimation mechanisms. Controlled front-end comparisons further show that CLAHE has its largest effect on the low-illumination sequences. Dynamic-region feature rejection reduces localization error on the easy dynamic-interference sequence, but this effect does not persist on the hard sequence. York-Tank can thus reveal system-specific degradation sensitivity, relate method performance changes to target degradation factors, and distinguish improvements in continuous tracking range from improvements in localization accuracy as degradation becomes more severe. York-Tank-GT produces valid PnP poses for 100% of overhead-camera frames within the common acquisition intervals with a 95th-percentile frame-wise reprojection RMSE of 1.102 px. Its output remains continuous on hybrid-degradation sequences for which COLMAP reconstruction is incomplete or fails, providing reference trajectories for quantitative evaluation under degraded conditions.

The scope of York-Tank is limited by its controlled tank environment, motion scale, and scene complexity. It is therefore intended to complement rather than replace datasets collected in real marine environments. The current dataset does not cover water-quality degradations such as turbidity and backscatter, so the reported performance trends require further validation in turbid or strongly scattering environments. The tank setup could be extended by incrementally adding and mixing suspended particles, measuring the resulting turbidity with a calibrated meter, and keeping the scene layout and illumination settings fixed. The validation of York-Tank-GT focuses on observation consistency and trajectory usability, and its absolute trajectory accuracy remains to be quantified using an external metrological system. Future work will extend the dataset to larger-scale scenes and will further develop independent accuracy validation for the reference trajectories. A preview release is available at https://doi.org/10.5281/zenodo.20432375, and the complete dataset and associated tools will be released at https://github.com/zhu-chengdong/York-Tank (accessed on 5 October 2026) after acceptance.

## Figures and Tables

**Table 1:** Comparison of representative underwater visual datasets across design dimensions relevant to SLAM evaluation.

Dataset	Acquisition Setting	Dominant-Factor Organization	Difficulty Organization	Repeatable Controlled Setup	Reference-Trajectory Source
Caves	Natural underwater caves	No	No explicit stratification	No	Underwater landmarks + 1-D ranging
AQUALOC	Real-world underwater environments	No	No explicit stratification	No	SfM/COLMAP
Eiffel Tower	Deep-sea structure	No	No explicit stratification	No	SfM/COLMAP
HAUD	Controlled tank	No	No explicit stratification	Yes	Motion capture
TANK	Controlled tank	No	Coupled-factor levels	Yes	Underwater AprilTag + AQUA-SLAM odometry
Ours	Controlled tank	Yes	Within-factor severity + hybrid conditions	Yes	Overhead camera + above-water AprilTag array

Note: Dominant-factor organization denotes a sequence-group design in which one degradation factor is treated as the primary controlled variable while the underlying scene layout and other acquisition conditions are kept as consistent as possible.

**Table 2:** Main specifications of the onboard stereo imaging unit.

Parameter	Value or Configuration
Camera model	Arducam OV9281 stereo camera
Resolution and frame rate	1280×800 pixels at 20 Hz per camera
Field of view	$82^{\circ }$ (D) × $70^{\circ }$ (H) × $55^{\circ }$ (V)
Shutter	Global shutter
Synchronization	Hardware synchronization
Stereo baseline	Approximately 0.101 m
Lighting control	Independent PWM control of the left and right lights

**Table 3:** Main specifications of the overhead monocular camera.

Parameter	Value or Configuration
Camera model	Arducam OV2311 monocular camera
Resolution and frame rate	1600×1300 pixels at 20 Hz
Field of view	$83^{\circ }$ (H)
Shutter	Global shutter

**Table 4:** Experimental settings for the baseline, easy, and hard single-degradation conditions.

Degradation	Control Variable	Baseline	Easy	Hard
Low illumination	Left/right light PWM	1600, 1600	1400, 1400	1200, 1200
Non-uniform illumination	Left/right light PWM	1600, 1600	1400, 1600	1400, 1800
Fast motion	Robot motion	Normal translation	Increased translation speed	Further increased translation speed
Texture deficiency	Primary viewing region	Normally textured regions of the base scene	Regions containing fewer textured objects	Primarily the textureless wall, with only occasional views of other objects
Dynamic interference	Dynamic-object configuration	No moving object	Moving toy shark with a smaller image footprint	Moving toy shark with a larger image footprint

Note: PWM denotes pulse-width modulation. The paired values are lighting-controller commands for the left and right lights, respectively.

**Table 5:** Quantitative characterization of the baseline, easy, and hard single-degradation conditions.

Dominant Degradation	Indicator	Level 1: Baseline	Level 2: Easy	Level 3: Hard
Low illumination	Median mean intensity *B*	29.97	21.36	9.10
Non-uniform illumination	Illumination spatial range *U*	19.91	63.52	91.10
Fast motion	Linear speed $V_{90}$	0.093 m/s	0.120 m/s	0.158 m/s
Texture deficiency	Normalized gradient $T_{n}$	0.505	0.350	0.194
Dynamic interference	Dynamic-region area $D_{95}$	0	24.53%	33.53%

**Table 6:** Composition and scale of the York-Tank test sequences.

Sequence	Dominant or Combined Degradation	Difficulty	Stereo Pairs	Duration (s)
1_reference1	Baseline	Level 1	1339	66.9
2_reference2	Baseline	Level 1	1235	61.7
3_dark_easy	Low illumination	Level 2	1234	61.6
4_dark_hard	Low illumination	Level 3	1452	72.5
5_uneven_easy	Non-uniform illumination	Level 2	1237	61.8
6_uneven_hard	Non-uniform illumination	Level 3	1179	58.9
7_fastmotion_easy	Fast motion	Level 2	1157	57.8
8_fastmotion_hard	Fast motion	Level 3	1330	66.4
9_fewtexture_easy	Texture deficiency	Level 2	1184	59.1
10_fewtexture_hard	Texture deficiency	Level 3	1310	65.4
11_dynamic_easy	Dynamic interference	Level 2	1151	57.5
12_dynamic_hard	Dynamic interference	Level 3	1205	60.2
13_hybrid	Dynamic interference + fast motion	Level 4	902	45.0
14_hybrid	Dynamic interference + low illumination	Level 4	1198	59.8
15_hybrid	Low illumination + dynamic interference + texture deficiency	Level 4	931	46.5
16_hybrid	Low illumination + dynamic interference + non-uniform illumination + fast motion + texture deficiency	Level 4	904	45.1
Total			18,948	946.2

**Table 7:** Evaluation results of ORB-SLAM3 and SVO Pro on the York-Tank test sequences.

Sequence	ORB-SLAM3 (Stereo)		SVO Pro (Stereo)	
	ATE RMSE (m)	Completeness	ATE RMSE (m)	Completeness
1_reference1	0.045902	99.85%	0.048684	95.74%
2_reference2	0.041106	99.76%	0.042199	96.52%
3_dark_easy	0.027366	61.26%	N/A	0%
4_dark_hard	N/A	0%	N/A	0%
5_uneven_easy	0.047024	99.76%	0.036530	79.55%
6_uneven_hard	0.065677	99.82%	0.051196	92.73%
7_fastmotion_easy	0.054671	99.83%	0.047473	56.09%
8_fastmotion_hard	0.075001	99.85%	0.060010	54.06%
9_fewtexture_easy	0.045738	56.91%	0.038363	51.22%
10_fewtexture_hard	0.049837	40.89%	0.053012	15.69%
11_dynamic_easy	0.073617	98.09%	0.071644	97.83%
12_dynamic_hard	0.056703	53.53%	0.107868	86.97%
13_hybrid	0.066903	60.20%	0.068424	32.71%
14_hybrid	0.131429	67.61%	0.092693	71.79%
15_hybrid	0.071264	21.70%	0.074616	23.31%
16_hybrid	0.105884	15.93%	0.088954	31.75%

Note: ATE denotes absolute trajectory error; RMSE denotes root mean square error. N/A indicates that the system produced no valid trajectory segment satisfying the requirements in Section 4.2.

**Table 8:** Evaluation results of ORB-SLAM3 and the two controlled front-end configurations on the York-Tank test sequences.

Sequence	ORB-SLAM3		ORB-SLAM3 + CLAHE		ORB-SLAM3 + YOLO	
	ATE RMSE (m)	Completeness	ATE RMSE (m)	Completeness	ATE RMSE (m)	Completeness
1_reference1	0.045902	99.85%	0.046843	99.85%	0.047057	99.85%
2_reference2	0.041106	99.76%	0.042690	99.68%	0.040974	99.68%
3_dark_easy	0.027366	61.26%	0.033279	88.82%	0.027302	61.02%
4_dark_hard	N/A	0%	0.029591	76.38%	N/A	0%
5_uneven_easy	0.047024	99.76%	0.048553	99.76%	0.047662	99.76%
6_uneven_hard	0.065677	99.82%	0.066470	99.91%	0.066260	99.82%
7_fastmotion_easy	0.054671	99.83%	0.055527	99.74%	0.054527	99.83%
8_fastmotion_hard	0.075001	99.85%	0.075977	99.70%	0.074659	99.85%
9_fewtexture_easy	0.045738	56.91%	0.047836	57.72%	0.045708	56.91%
10_fewtexture_hard	0.049837	40.89%	0.047524	39.54%	0.049048	40.81%
11_dynamic_easy	0.073617	98.09%	0.090399	99.83%	0.058532	99.74%
12_dynamic_hard	0.056703	53.53%	0.057553	53.44%	0.056484	53.44%
13_hybrid	0.066903	60.20%	0.044612	49.33%	0.056994	91.24%
14_hybrid	0.131429	67.61%	0.134819	89.57%	0.131012	67.61%
15_hybrid	0.071264	21.70%	0.081649	33.40%	0.079075	27.60%
16_hybrid	0.105884	15.93%	0.102938	43.81%	0.105788	15.93%

**Table 9:** Grouped mean performance changes of the two front-end configurations relative to the ORB-SLAM3 baseline.

Dominant Degradation Condition	CLAHE Completeness Change (pp)	CLAHE ATE Change (%)	YOLO Completeness Change (pp)	YOLO ATE Change (%)
Low illumination	+51.97	+21.61 *	−0.12	−0.23 *
Non-uniform illumination	+0.05	+2.06	0.00	+1.08
Fast motion	−0.12	+1.41	0.00	−0.37
Texture deficiency	−0.27	−0.23	−0.04	−0.86
Dynamic interference	+0.83	+13.53	+0.78	−11.74
Hybrid degradation	+12.67	−3.05	+9.24	−0.70

Note: “+” and “−” denote an increase and decrease relative to the baseline, respectively. An asterisk indicates an ATE change calculated from the easy low-illumination sequence only; the ORB-SLAM3 baseline failed on the hard sequence, which is therefore excluded from the mean ATE calculation.

**Table 10:** Mean performance of ORB-SLAM3 and SVO Pro at each difficulty level.

Difficulty	ORB-SLAM3		SVO Pro	
	Completeness	ATE RMSE (m)	Completeness	ATE RMSE (m)
Level 1	99.81%	0.043504	96.13%	0.045442
Level 2	83.17%	0.049683	56.94%	0.048503
Level 3	58.82%	0.061805	49.89%	0.068022
Level 4	41.36%	0.093870	39.89%	0.081172

Note: Mean trajectory completeness includes failed sequences with zero completeness; mean ATE RMSE includes only sequences with valid trajectories.

**Table 11:** AprilTag observation and PnP statistics for York-Tank-GT.

Evaluation Metric	Result
Proportion of valid PnP poses	100%
Mean visible AprilTags per frame	8.90
95th percentile of frame-wise reprojection RMSE	1.102 px

**Table 12:** Consistency between COLMAP reconstructions and York-Tank-GT left-camera reference trajectories on representative sequences.

Sequence	Difficulty Level	COLMAP Reconstruction Completeness	COLMAP vs. York-Tank-GT ATE RMSE (m)
1_reference1	Level 1	100%	0.0419
5_uneven_easy	Level 2	100%	0.0451
8_fastmotion_hard	Level 3	100%	0.0721
14_hybrid	Level 4	78.55%	0.0788
16_hybrid	Level 4	0%	N/A

## Data Availability

A preview release containing the first baseline sequence is publicly available on Zenodo at https://doi.org/10.5281/zenodo.20432375. The complete York-Tank dataset, calibration files, reference trajectories, and evaluation tools will be made publicly available at https://github.com/zhu-chengdong/York-Tank after acceptance.

## Abbreviations

The following abbreviations are used in this manuscript:

6-DoF	Six degrees of freedom
ATE	Absolute trajectory error
CLAHE	Contrast-limited adaptive histogram equalization
IPPE	Infinitesimal plane-based pose estimation
PnP	Perspective-n-point
PWM	Pulse-width modulation
RMSE	Root mean square error
ROS	Robot Operating System
SfM	Structure from motion
SLAM	Simultaneous localization and mapping
USBL	Ultra-short baseline
YOLO	You Only Look Once
